# The Association between ADL Ability and Quality of Life among People with Advanced Cancer

**DOI:** 10.1155/2019/2629673

**Published:** 2019-09-02

**Authors:** Mette Falk Brekke, Karen la Cour, Åse Brandt, Hanne Peoples, Eva Ejlersen Wæhrens

**Affiliations:** ^1^Department of Occupational Therapy, University College Absalon, Næstved, Denmark; ^2^Department of Public Health, Research Unit for General Practice, University of Southern Denmark, Odense, Denmark; ^3^The Danish Knowledge Center for Rehabilitation and Palliative Care, REHPA, Vestergade 17, 5800 Nyborg, Denmark; ^4^Department of Public Health, Research Unit for General Practice, The Research Initiative for Activity Studies and Occupational Therapy, University of Southern Denmark, Odense, Denmark; ^5^Centre for Disability and Mental Vulnerability, The National Board of Social Services, Odense, Denmark; ^6^Faculty of Health Sciences, Health Sciences Research Center, University College Lillebaelt, Odense, Denmark; ^7^The Parker Institute, Copenhagen University Hospital Bispebjerg & Frederiksberg, Copenhagen, Denmark

## Abstract

**Background:**

Occupational therapy and occupational science are founded on the theoretical core assumption that occupation and quality of life (QoL) are closely related. However, such theoretical core assumptions must be supported through empirically based research.

**Objective:**

To investigate the association between QoL and occupation, here self-reported and observed ADL abilities as a part of occupation, among people with advanced cancer, including determining whether self-reported or observed ADL ability had the stronger association with QoL.

**Methods:**

The study was nested in a cross-sectional study. The association between ADL ability and QoL among 108 people with advanced cancer was investigated using the ADL Interview (ADL-I), the Assessment of Motor and Process Skills (AMPS), and the European Organization for Research and Treatment of Cancer Core Quality of Life Questionnaire (EORTC-QLQ-C30).

**Results and Conclusions:**

Results showed that high observed ADL motor ability was associated with high QoL. In contrast, observed ADL process ability and self-reported ADL ability were not significantly associated with QoL. Oppositely expected, observed ADL ability had a stronger association with QoL than self-reported ADL ability. Thereby, the study to some extent contributes knowledge confirming the theoretical core assumptions about the relation between occupation, here performance of ADL, and QoL.

## 1. Introduction

Occupational therapy (OT) and occupational science (OS) are founded on the theoretical core assumption that occupation and quality of life (QoL) are closely related [1–4]. Occupation can be defined as “a person's engagement in a process,” which takes place in meaningful and purposeful doing for the individual [5]. Occupation is seen as a basic human need and as a means to promote QoL [1–4]. Based on this theoretical core assumption, the main purpose of OT is to support people in occupation, i.e., participation in daily life tasks they find meaningful and necessary to do or are expected to do in their everyday lives [5]. Necessary and relevant tasks comprise, amongst others, activities of daily living (ADL) [6], including basic, personal self-care tasks; personal ADL (PADL) and more complex household tasks; and instrumental ADL (IADL). While PADL and IADL tasks are crucial for independent functioning and living at home, it is individually determined whether participation in such ADL tasks is perceived as a meaningful and purposeful occupation [6].

Likewise, the concept of QoL is understood as individually determined and influenced by the context in which the person lives [1]. There are several understandings of QoL, including the one offered in the theories by Townsend and Polatajko (2007) [1]: “Quality of life from an occupational perspective, refers to choosing and participating in occupations that foster hope, generate motivation, offer meaning and satisfaction, create a driving vision of life, promote health, enable empowerment, and otherwise address the quality of life” [1]. This definition implies that QoL is a multidimensional phenomenon, as many factors are part of and influence QoL. However, to the best of our knowledge, there are not developed instruments to assess QoL based on this definition. Therefore, it is relevant to involve related ways to complement the understanding and measuring of QoL. Within healthcare, it has become common to focus on assessing QoL, to capture the patients' perception of QoL in relation to their state of health [7]. For example, the European Organization for Research and Treatment of Cancer (EORTC) uses the term health-related quality of life (HRQOL), when assessing patients' symptoms, functioning, and overall well-being by self-report [8]. The EORTC understands HRQOL as a multi-dimensional construct covering at least several key dimensions such as disease and treatment-related symptoms as well as physical, psychological and social functioning [8]. Both definitions imply an understanding of QoL as a multidimensional construct and as individually determined [1, 8]. However, when the definition offered by Townsend and Polatajko captures a person's QoL in a wide perspective [1], the definition by the EORTC is more narrowly related to a person's state of health [8].

Within the framework of evidence-based practice, it is essential that the theoretical core assumption about a relationship between occupation and QoL is supported by empirically based research [3, 9]. Studies have documented that people with a wide range of chronic illnesses experience limited ADL ability [10] and reduced QoL [11–14]. This research includes people with advanced cancer, defined as an incurable cancer diagnosed by an oncologist, reporting long-term or permanent problems with decreased ADL ability [15–20], as well as reduced QoL [17]. For example, Johnsen et al. [18] conducted a study involving 997 people with advanced cancer and found that 48% of the participants reported problems with ADL and work and 29% had unmet needs concerning these problems. Cheville et al.'s study of women with metastatic breast cancer (*n* = 163) showed that 43% had difficulties with PADL and 74% regarding IADL [19]. Furthermore, studies involving persons with cancer, including advanced cancer, have documented a temporal gradual deterioration in ADL ability as well as a decline in QoL [21–23].

Interestingly, Solomon et al. [24] reported, in a cohort study based on interviews with elderly persons with advanced illness (*n* = 158) (29% were cancer patients), that both ADL ability and depressed mood were associated with QoL. This conclusion is supported by Yokoo et al. who, in an internet-based questionnaire, found in persons with cancer (*n* = 807) that “concerns about daily living” were one of many factors significantly associated with QoL [25]. This is also consistent with findings in a study on elderly persons with cancer in hospice (*n* = 533). Based on interviews, they concluded that “functional status,” defined as the ability to perform basic activities important to maintain independence, was one predictor of QoL [26]. Thus, based on evaluations of ADL ability using self-report, i.e., questionnaires and interviews, there seems to be some evidence to support the theoretical assumption about a relation between occupation, here performance of ADL, and QoL.

However, when evaluating ADL ability, studies recommend applying a combination of self-reporting and observation, as the two methods seem to provide complimentary information about ADL ability [27–29]. Therefore, it is a limitation that current studies about ADL and its association with QoL are based on self-reported data only. Furthermore, there is evidence of a stronger relationship between self-reported data in general and data based on self-reporting and observation [30], indicating that information from the same source, e.g., the person self-reporting, will have a stronger association than information from different sources. Thus, it would also be relevant to investigate whether self-reported ADL ability has a stronger association with QoL than observed ADL ability.

The aim of this study was, therefore, to investigate the association between QoL and occupation, here self-reported and observed ADL abilities, as a part of occupation among people with advanced cancer, including determining whether self-reported or observed ADL ability had the stronger association with QoL.

The hypotheses were according to the theoretical core assumptions that (a) self-reported ADL ability would be statistically significantly associated with QoL (*P* ≤ 0.05), (b) observed ADL ability would be statistically significantly associated with QoL (*P* ≤ 0.05), and (c) self-reported ADL ability would have a stronger association with QoL than observed ADL ability in people with advanced cancer (*P* ≤ 0.05).

## 2. Materials and Methods

### 2.1. Participants

This paper is based on data retrieved in a cross-sectional study as part of a larger project: “Activity, Cancer, and Quality of Life at Home” (the ACQ Project) [31, 32]. Participants in that study were recruited among outpatients from oncology units at Odense and Aarhus University Hospitals in Denmark from January 2013 to April 2014. The inclusion criteria for the study were as follows: ≥18 years old, diagnosed with disseminated cancer; receiving palliative interventions in the respective outpatient units; estimated survival time of at least one month; WHO performance status 1-3; living at home or in sheltered living; and able to fill in questionnaires and participate in interviews.

Data from 164 participants were contained in the study database. The most common types of cancer among the participants were cancer in the lungs, colon/rectum, breast, and prostate. For the present study, only participants with complete data according to the variables in the study were included. Thus, 39 participants with incomplete data sets were removed, due to missing WHO performance status, ADL, and/or QoL data, leaving a total number of 125 participants.

### 2.2. Design and Procedures

The data collection in the cross-sectional study included a combination of study-specific and standardized questionnaires: standardized semistructured interviews and standardized observations. The participants received the questionnaires by mail. These were followed up by a home visit within one week. Four trained data collection occupational therapists (D-OTs) conducted the home visits and the data collection including performing the interviews and the observations according to a data collection manual.

### 2.3. Instrumentation

The questionnaires were twofold. The first part was a basic information questionnaire with 11 items regarding sociodemographic information, such as gender, age, and living alone/living in cohabitation. The second part was the European Organization for Research and Treatment of Cancer (EORTC) Core Quality of Life Questionnaire (QLQ-C30). The instruments applied during the home visits included the ADL Interview (ADL-I) and the Assessment of Motor and Process Skills (AMPS).

### 2.4. European Organization for Research and Treatment of Cancer Core Quality of Life Questionnaire (EORTC-QLQ-C30)

The EORTC-QLQ-C30 (v. 3.0) [33, 34] is a standardized cancer-specific questionnaire measuring health-related quality of life. Its psychometric properties have been investigated in several studies on cancer patients, including patients with advanced cancer, and it is found to be a valid and reliable instrument [33, 35–38]. The EORTC-QLQ-C30 consists of 30 items addressing five multi-item scales of functioning (physical, role, social, emotional, and cognitive functioning), three multi-item scales (fatigue, nausea and vomiting, and pain), and six single items (dyspnoea, constipation, sleeping problems, appetite loss, diarrhoea, and financial problems) related to symptoms or problems. Furthermore, it includes a combined scale with two items, one addressing global health and another global QoL, both being answered on a seven-point ordinal scale (1 = very poor, 7 = excellent). In the current study, the item addressing global quality of life (EORTC QoL): “How would you rate your overall quality of life during the past week?” was used to get a pure expression of QoL [34].

### 2.5. The ADL Interview (ADL-I)

The ADL Interview (ADL-I) [28, 39] is a standardized OT evaluation tool developed to describe and measure the quality of ADL task performance based on self-report. When using the ADL-I, the person is requested to evaluate the quality of his or her performance in 47 ADL tasks: 31 tasks related to personal ADL (PADL) and 16 tasks related to instrumental ADL (IADL). The D-OT instructs the person to rate the quality of the task performance, where PADLs are based on the last 24 hours and IADLs are based on the last week. The person reports his or her ability using seven response categories reflecting efficiency, effort/fatigue, safety, and independence [28, 39].

Categorical data from the ADL-I are converted into an overall linear measure of each person's self-reported quality of ADL task performance presented in logits (log-odds probability units) based on Rasch measurement methods [28, 39]. A higher measure indicates more ADL ability. It represents each person's self-reported overall quality of ADL task performance, adjusted for the difficulty of the ADL tasks. [39]. Studies support that the ADL-I can generate valid and reliable linear measures of self-reported quality of ADL task performance in persons with long-term chronic diseases [12, 27, 28, 39] including advanced cancer [40].

### 2.6. Assessment of Motor and Process Skills (AMPS)

The Assessment of Motor and Process Skills (AMPS) [41, 42] is a standardized observation-based evaluation tool used by OTs to measure a person's observed quality of ADL task performance regarding physical effort and/or fatigue, efficiency, safety, and independence. When the AMPS is applied, the calibrated and trained OT is observing the person, while he or she is performing at least two relevant and familiar standardized ADL tasks of their own choice [42]. The OT is observing the quality of 16 motor and 20 process performance skills, and thereafter, the OT evaluates the quality of each skill on a four-point ordinal scale in accordance with the scoring criteria in the AMPS manual [42]. Finally, the ordinal scores are transformed into overall linear measures of ADL motor and ADL process abilities, respectively, by using the AMPS 9.0 software [43], based on a many-faceted Rasch measurement model. The measures are thereby adjusted for task challenge, skill item difficulty, and rater severity. The two measures are expressed in logits, where higher measures indicate more ADL ability [41]. Several studies have found the AMPS ability measures valid and reliable across age, gender, and diagnostics groups [41], including cancer [44, 45].

### 2.7. Ethical Considerations

The project was registered and approved by the Danish Data Protection Agency (J.nr. 2012-41-1404). According to the Regional Committees on Health Research Ethics for Southern Denmark, the ACQ project was not notifiable (Project ID S-20122000-96-CKH/csf). The principles of the Helsinki Declaration were followed [46]. Thereby, the potential participants received verbal and written information about the purpose of the study, confidentiality, and the right to withdraw from the study. All the participants signed a written consent form. The data was handled in accordance with the aim of the research project, and the data has been administrated respectfully and confidentially.

### 2.8. Data Analysis

The statistical analyses were performed using the statistical software package, the Stata software (v.14.1) by the StataCorp [47]. The categorical descriptive data: gender, living alone or in cohabitation, and EORTC QoL, were presented based on numbers and percentages. The interval scale data were tested for normal distribution, first visually with a QQ-plot and a histogram and then tested with a Shapiro Wilk test [48, 49]. Due to lack of normal distribution and with a primarily left skewed distribution, age as well as the measures of ADL ability was analysed using nonparametric statistics and presented based on median and range [48]. In addition, in the descriptive part, the variables were also analysed and presented based on numbers and percentages. This is due to the categorization of the interval scale measures of ADL ability in the regression analyses. Further, the process of categorization will be elaborated later in this section.

As the outcome variable, EORTC QoL, was rated on an ordinal scale, nonparametric statistics based on ordered logistic regression analysis were used to investigate ADL ability as a predictor of QoL [48, 49]. However, there were very low numbers (<8) to fit the model in the first, second, and seventh categories for it to be included in the ordered logistic regression analysis model with seven categories for outcome. Therefore, only answers distributed in categories three to six on the EORTC QoL scale were included in the analysis.

The ADL-I overall linear measure of self-reported quality of ADL task performance, the overall linear AMPS ADL motor ability measures, and the overall linear AMPS ADL process ability measures were chosen as exposure variables. All three exposure variables where each categorized into three subgroups [48]. Since there in the literature were no cut-offs for the interpretation of the ADL-I measures, the ADL-I data were categorized to ensure equal distribution of participants in each of three subgroups [48]: low ADL ability < 1.64 logits, middle ADL ability ≥ 1.64 to <2.37 logits, and high ADL ability ≥ 2.37 logits.

The criteria for categorization of the AMPS ADL motor and ADL process ability measures, respectively, were based on the independence cut-offs, predicting a need for assistance [41, 50, 51]. Therefore, the overall linear AMPS ADL motor ability measures were categorized into the following: low ADL ability < 1.0 logits, middle ADL ability ≥ 1.0 to <1.5 logits, and high ADL ability ≥ 1.5 logits. Likewise, the overall linear AMPS ADL process ability measures were categorized into the following: low ADL ability < 0.7 logits, middle ADL ability ≥ 0.7 to <1.0 logits, and high ADL ability ≥ 1.0 logits [41, 50, 51].

To address the first aim in the study, a crude model of ordered logistic regression analysis [48, 49] was conducted for each of the three exposure variables with the EORTC QoL as the outcome. The assumptions for the goodness of fit in the ordered logistic regression models were likewise tested for each of the three models. The test addressed whether the outcome variable was ordered and if the effects of the exposures were alike for all categories in the outcome variable [48, 49].

Demographic data including gender, age, and living alone or in cohabitation were selected to control for confounding in an adjusted model of the ordered logistic regression analysis [48, 49]. This was to examine if they influenced the association between ADL ability and QoL [17, 52, 53]. As studies report opposing results about the influence of cohabitation on persons' report on symptoms [17, 53–55], it is also relevant to examine whether living alone or in cohabitation has influence. First, age was categorized into two groups: <65 years and >65 years old [48] Next, ordered logistic regression analyses were conducted, for each of the three potential confounders, to determine if they were associated with QoL. If estimates were significant, the following adjusted models of the regression analyses were planned: one adjusted model to control for age and gender and a second adjusted model to control for the first model as well as living alone or not.

The estimates in the ordered logistic regression analyses were presented based on the odds ratios (ORs) with 95% confidence intervals (CI) and a statistical significant *P* value at <0.05 [48, 49]. The ORs are being interpreted as the exposure odds ratios of being in the higher-category outcome for the EORTC QoL in comparison with the lower-category outcome [48]. Finally, in the crude analysis, graphs of the probability of the association between ADL ability and QoL were developed for each of the three exposure variables [49].

In order to address the second and last aim of the study, to test whether self-reported ADL ability would have a stronger association with QoL than observed ADL ability, a *z*-test for a difference in two odds ratios was applied to investigate a statistically significant difference among the three exposure variables on EORTC QoL [48, 49]. First, the ORs for middle ADL-I ability were tested with a *z*-test, for the difference between the ORs for AMPS motor middle ADL ability as well as for AMPS process middle ADL ability, respectively. Likewise, the OR for high ADL-I ability were tested for the difference between the ORs for AMPS motor high ADL ability as well as for AMPS process high ADL ability, respectively. The estimates were presented based on the corresponding *P* values according to the *z*-scores, with a statistical significance at *P* < 0.05 [48].

## 3. Results

Due to the low number of participants in the first, second, and seventh categories of the EORTC, additionally, 17 participants were excluded, leaving in total 108 participants for the analyses. The participants were distributed in WHO performance status (PS) categories 1-3: PS 1 (*n* = 62), PS 2 (*n* = 43), and PS 3 (*n* = 3). The remaining descriptive characteristics and ADL ability measures are presented in Table 1, for the total group and by EORTC QoL categories three to six, respectively. As illustrated, the 108 participants were aged 36 years or older, with two-thirds living in cohabitation and with an almost equal gender distribution. Most participants (30%) rated their QoL in the EORTC QoL category four, which pertains to the middle of the scale, and the majority of these were women (64%). The fewest participants (18%) rated their QoL in the better end of the scale (category six), but in contrast to the lower categories of QoL, the vast majority of these were men (79%). In the group, category six, the majority also had high ADL ability, both self-reported and observed (Table 1). The majority of the participants, who rated EORTC QoL in the lower end (category three), overall had low AMPS ADL motor ability, whereas their ADL-I and AMPS ADL process ability measures represented more variation (Table 1).

### 3.1. The Association between ADL Ability and QoL

The assumptions for the goodness of fit for the ordered logistic regression models were tested and found statistically acceptable. Results, as presented in Table 2, show for the crude model that self-reported ADL ability, as measured by ADL-I, was not associated with QoL. In contrast, results indicate that observed ADL ability was significantly associated QoL. Thus, in the subgroups with high ADL ability, both the AMPS ADL motor (*P* = 0.02) and AMPS ADL process (*P* = 0.03) abilities were significantly associated with a higher-category QoL (Table 2).

**Figure 1:**
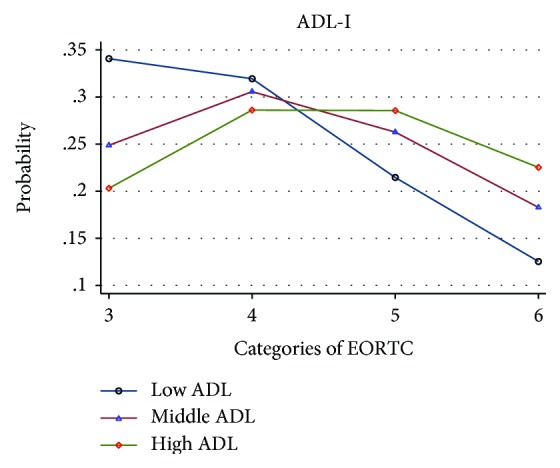
Probability of the association between ADL-I and EORTC QoL, categorized into low, middle and high ADL abilities.

**Figure 2:**
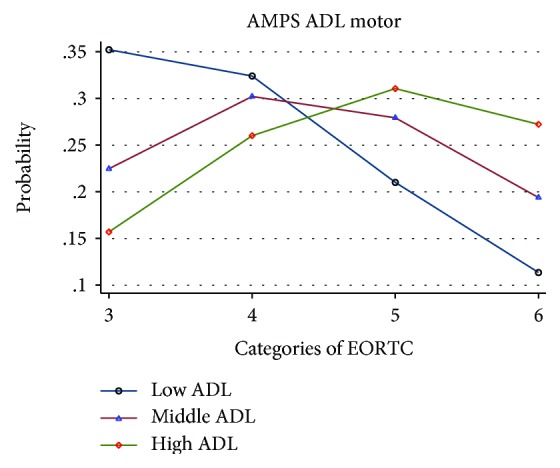
Probability of the association between AMPS ADL motor and EORTC QoL, categorized into low, middle and high ADL abilities.

**Figure 3:**
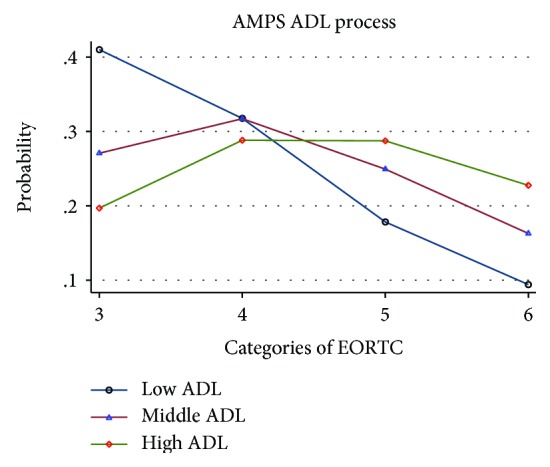
Probability of the association between AMPS ADL process and EORTC QoL, categorized into low, middle and high ADL abilities.

When viewing Figures 1–3, illustrating the probability of an association between ADL ability and EORTC QoL categories three to six, there is a trend towards that both self-reported and observed ADL abilities were associated with QoL. For all exposures (ADL-I [Figure 1], AMPS ADL motor [Figure 2], and AMPS ADL process [Figure 3]), participants with a high ADL ability had the largest probability to rate a better QoL and the lowest probability to rate a poorer QoL. In contrast, participants with a low ADL ability had the largest probability to rate a poorer QoL and the lowest probability to rate a better QoL.

In the ordered logistic regression analysis for each of the potential confounders, only the estimate for gender was significant (*P* = 0.02) (Table 3) and thereby the only one included to control for confounding in the adjusted regression model. In the adjusted regression analyses (Table 2), the results for ADL-I remained nonsignificant. In the group with high ADL ability, the AMPS ADL motor remained significantly associated with QoL (*P* = 0.04). For the AMPS ADL process, the adjustment for gender resulted in a borderline significant association with QoL in the group with high ADL ability (*P* = 0.053).

### 3.2. The Stronger Association with QoL

Results related to the second aim, testing whether self-reported ADL ability would have a stronger association with QoL than observed ADL ability, are shown in Table 4. When comparing ORs for self-reported and observed ADL abilities and its association with QoL, there were no statistically significant differences between the tested ORs: neither between the ORs for ADL-I and AMPS motor nor between the ORs for ADL-I and AMPS process for subgroups with middle or high ADL ability (Table 4). Thereby, self-reported ADL ability did not have a stronger association with QoL than observed ADL ability in people with advanced cancer.

## 4. Discussion

The overall purpose of this study was to investigate the association between QoL and occupation, here self-reported and observed ADL abilities as part of occupation, among people with advanced cancer, including determining whether self-reported ADL ability would have a stronger association with QoL than observed ADL ability.

The study results confirm the hypothesis of an association between observed ADL ability and QoL, in a subgroup of participants with high ADL motor ability. In contrast, observed ADL process ability and self-reported ADL ability were not significantly associated with QoL. Thus, the study results to some extent support the theoretical core assumption of OT and OS and a relationship between occupation, here ADL, and QoL [1–4]. Townsend and Polatajko [1] argue that QoL for all people are highly affected by the occupations they are engaged in. Therefore, enabling occupation is the key point in their vision for OT [1]. Similarly, Kielhofner [3] also highlighted the importance of occupation for QoL as a core value within the present OT paradigm. Furthermore, he stressed that people with limited access to participation in occupations can experience a decrease in QoL [3].

From an OS perspective, Yerxa described the close relationship between occupation and QoL [2, 4], including how engagements in diverse daily occupations are part of shaping the individual's perceived QoL [4]. Yerxa highlighted the OT's role, helping people with chronic diseases to obtain fulfilment in their ADL and thereby promote QoL [2]. Therefore, this theoretical core assumption of OT and OS also has its relevance for people with advanced cancer.

That ADL ability was associated with QoL among people with advanced cancer was partly in line with previous studies [24–26]. As mentioned earlier, a study found that ADL ability was associated with QoL in elderly persons with advanced illness [24]. Likewise, another study concluded “concerns about daily living” among people with cancer were significantly associated with QoL [25]. Finally, “functional status” defined as the ability to perform basic activities important to maintain independence was a predictor of QoL among elderly persons with cancer in hospice [26] However, the results in these studies were based on self-reported ADL ability only. In contrast, the current study only found a significant association between observed ADL motor ability and QoL. Another difference was that the previous studies assessed 2-6 items of ADL and only in terms of independence in ADL. [24–26]. In comparison, the current study included a broader range of PADL and IADL tasks evaluated in relation to the overall quality of performance, using both self-report and observation-based ADL instruments [28, 39, 41, 42]. Therefore, by applying the ADL-I and AMPS instruments, this study offered a more informed perspective on the person's ADL ability and its association with QoL.

Another reason for differences between study results could be the instruments used to assess QoL. In the present study, the single item from the EORTC-QLQ-C30 was chosen. A similar approach was used in the study involving elderly persons with advanced illness [24] by posing one question asking participants to rate their overall QoL. In contrast, in the two studies involving persons with cancer, a somewhat broader approach for assessing QoL was employed by using the Global Health Status score of the EORTC-QLQ-C30 [25] and the Hospice Quality of Life Index-14 [26], respectively.

Another essential finding was the rejection of the third hypothesis regarding self-reported ADL ability having the stronger association with QoL. These results are in contrast to findings in the study by Amris et al. [30], who found a stronger relationship between self-reported data in general than between self-reported and observed data. One reason for this difference could be that the study by Amris et al. involved variables like pain and psychological distress (e.g., anxiety and catastrophizing), not ADL ability [30].

### 4.1. Strength and Limitations of the Study

One strength of this study is that the recruitment of participants was carried out using explicit inclusion criteria [56]. Further, it is considered a strength that all data were collected using standardized instruments validated among people with advanced cancer and with explicit procedures for data collection carried out by trained D-OTs, minimizing the risk of information bias in the study. Furthermore, using two instruments based on self-report and observation, respectively, to measure ADL ability is considered a strength, as previous studies recommend applying both methods in evaluating ADL ability [27, 28].

Moreover, in this study, one of the two items from the combined scale addressing global health and overall QoL in the EORTC-QLQ-C30 was chosen: “How would you rate your overall quality of life during the past week?” The reason for using a single item from an established scale was to ensure the evaluation of the participants' perceived overall QoL without simultaneously influence from their perceived global health. Though this may be considered a potential limitation to the study results, this choice is well-founded. QoL is a complex concept to assess, as many aspects of life, including health status, may influence a person's perceived QoL. While the application of the combined scale of the global health and QoL in the EORTC-QLQ-C30 questionnaire might have provided more information about QoL, it would still not embrace all potential elements to a comprehensive study of a person's QoL. For example, as mentioned earlier, the understanding of QoL from an OT perspective by Townsend and Polatajko includes elements like choosing and participating in occupations that foster hope and generate motivation [1]. In the absence of a tool incorporating such aspects, we therefore believe that using a single question addressing the participant's perceived QoL is a better choice.

The study showed a statistically significant association between observed ADL motor ability and high QoL. However, observed ADL process ability and self-reported ADL ability were not significantly associated with QoL, even though there was a tendency towards those participants with a high self-reported and/or observed ADL ability also reporting a better QoL. Therefore, the sample size may be considered a limitation, since the lack of a statistical significance may be explained by a small number of participants within each of the response categories of the EORTC-QLQ-C30. Furthermore, the sample did not represent the full spectrum of the EORTC QoL scale, and consequently, the interpretation of the results must be with some caution.

When preparing AMPS and ADL-I data for data analysis, the AMPS motor and process ability measures were categorized based on established independence cut-offs. A similar approach was not possible, when handling the ADL-I measures, as cut-offs for interpretation of the ADL-I measures have not yet been established. This may be considered a study limitation, since there is a risk that the results could have been different, if other cut-points had been set. Still, the risk was considered minor, as the way to create the three ADL-I subgroups was plausible and acknowledged in the literature [41, 48, 50, 51].

### 4.2. Implications for Clinical Practice

Based on the study findings, it may be relevant for clinicians to suggest occupation-focused or occupation-based interventions as part of the palliative rehabilitation services, when working with people with severe diseases, like advanced cancer [5]. A relevant OT intervention could be an adaptive occupation-based intervention, focusing on diminishing the physical effort and fatigue during ADL task performance (e.g., assistive technology, energy conservation, and alternative methods of doing) [5, 57–59]. Nevertheless, it is important to bear in mind that it is individually determined whether participation in the performance of ADL tasks is perceived as a meaningful and purposeful occupation. Some people with advanced cancer may wish to prioritize time and energy in another ways. In addition, to ensure rehabilitation and palliative care services at the individual level, it is essential to gain knowledge of the clients' perspective on their experiences of ADL ability [1, 5, 59]. However, this study revealed an association between observed high ADL motor ability and QoL, which adds to the perspective that it is also of importance to focus on the professionals' perspective, using observation as enabled with, e.g., the AMPS. Recommendations regarding applying both self-report and observation when evaluating the quality of ADL task performance are in line with other studies [27, 28].

## 5. Conclusion

In conclusion, this study found that observed high ADL motor ability had a statistically significant association with high QoL among people with advanced cancer. In contrast, observed ADL process ability and self-reported ADL ability were not significantly associated with QoL. Furthermore, the study found self-reported ADL ability not having a stronger association with QoL than observed ADL ability. Thus, despite the potential limitations, the study added a piece of knowledge to the evidence confirming the theoretical core assumptions regarding a close relationship between occupation and QoL, as understood in OT and OS [1–4]. However, the knowledge is limited; that is why future studies on the association between occupation, here ADL ability, and QoL among people with long-term or chronic illnesses are recommended.

## Figures and Tables

**Table 1:** Summary of participant characteristics.

Characteristics	Total *n* = 108	EORTC 3^‡^ *n* = 29 (27)	EORTC 4^‡^ *n* = 33 (30)	EORTC 5^‡^ *n* = 27 (25)	EORTC 6^‡^ *n* = 19 (18)
Age, median (range)^1^	67 (36-89)	67 (38-86)	67 (36-82)	67 (43-80)	66 (55-89)
Gender					
Women, *n* (%)	56 (52)	17 (59)	21 (64)	14 (52)	4 (21)
Men	52 (48)	12 (41)	12 (36)	13 (48)	15 (79)
Living in cohabitation					
Yes, *n* (%)	70 (65)	19 (66)	21 (64)	18 (67)	12 (63)
No	38 (35)	10 (44)	12 (36)	9 (33)	7 (37)
ADL-I (logits)^1,2^					
Low [<1.64], *n* (%)	37 (35)	11 (38)	15 (45)	7 (26)	4 (21)
Low [<1.64], median [range]	1.06 [-0.74–1.64]				
Middle [1.64–2.37], *n* (%)	35 (32)	8 (28)	11 (33)	11 (41)	5 (26)
Middle [1.64–2.37], median [range]	2.04 [1.68–2.37]				
High [>2.37], *n* (%)	36 (33)	10 (34)	7 (22)	9 (33)	10 (53)
High [>2.37], median [range]	3.29 [2.46–6.22]				
AMPS ADL motor (logits)^1,2^					
Low [<1], *n* (%)	48 (44)	15 (52)	19 (58)	10 (37)	4 (21)
Low [<1], median [range]	0.53 [-2.04–0.99]				
Middle [1–1.5], *n* (%)	30 (28)	6 (21)	9 (27)	11 (41)	4 (21)
Middle [1–1.5], median [range]	1.31 [1.01–1.49]				
High [>1.5], *n* (%)	30 (28)	8 (27)	5 (15)	6 (22)	11 (58)
High [>1.5], median [range]	1.94 [1.53–2.52]				
AMPS ADL process (logits)^1,2^					
Low [<0.7], *n* (%)	22 (20)	9 (32)	7 (22)	4 (15)	2 (10)
Low [<0.7], median [range]	0.41 [-1.85–0.67]				
Middle [0.7–1], *n* (%)	40 (37)	10 (34)	14 (42)	10 (37)	6 (32)
Middle [0.7–1], median [range]	0.87 [0.71–0.98]				
High [>1], *n* (%)	46 (43)	10 (34)	12 (36)	13 (48)	11 (58)
High [>1], median [range]	1.14 [1–2.11]				

ADL-I: ADL Interview; AMPS: Assessment of Motor and Process Skills; ^‡^EORTC-QLQ-C30: European Organization for Research Treatment of Cancer Core Quality of Life Questionnaire. Item #30 “Global Quality of Life” is rated on an ordinal scale ranging from 1 (very poor) to 7 (excellent). The EORTC-scale categories 1, 2, and 7 are excluded due to very low numbers to fit in the statistical model. Data presented as the number (*n*) of subjects and percentage (%). ^1^Based on the median and range due to lack of normal distribution in data. ^2^Linear measures of self-reported (ADL-I) and observed (AMPS) qualities of ADL task performance expressed in Rasch-based logistically transformed units (logits).

**Table 2:** Ordered logistic regression analysis (*n* = 108) of the association between ADL ability and EORTC QoL^‡^.

Exposures	Crude model OR [95% CI]	*P* value	Adjusted model^a^ OR [95% CI]	*P* value
ADL-I				
Low	1		1	
Middle	1.56 [0.69–3.53]	0.29	1.70 [0.74–3.87]	0.21
High	2.03 [0.87–4.73]	0.10	1.93 [0.82–4.53]	0.13
AMPS ADL motor				
Low	1		1	
Middle	1.88 [0.84–4.19]	0.13	1.72 [0.77–3.88]	0.19
High	2.92 [1.21–7.05]	0.02	2.53 [1.03–6.17]	0.04
AMPS ADL process				
Low	1		1	
Middle	1.87 [0.73–4.83]	0.19	1.92 [0.74–4.98]	0.18
High	2.83 [1.1–7.27]	0.03	2.56 [0.99–6.65]	0.053

ADL-I: ADL Interview; AMPS: Assessment of Motor and Process Skills; OR: odds ratio; CI: confidence interval; ^‡^EORTC-QLQ-C30: European Organization for Research Treatment of Cancer Core Quality of Life Questionnaire. Item #30 “Global Quality of Life” is rated on an ordinal scale ranging from 1 (very poor) to 7 (excellent). The EORTC-scale categories 1, 2, and 7 are excluded due to very low numbers to fit in the statistical model. ^a^Adjusted model: adjusted for gender.

**Table 3:** Ordered logistic regression analysis (*n* = 108) of the potential confounders associated with EORTC QoL^‡^.

Potential confounders	Crude OR [95% CI]	*P* value
Gender		
Men	1	
Women	0.43 [0.21–0.86]	0.02
Age		
<65 year	1	
≥65 years	1.07 [0.53–2.14]	0.86
Living in cohabitation		
No	1	
Yes	0.98 [0.48–1.99]	0.96

OR: odds ratio; CI: confidence interval; ^‡^EORTC-QLQ-C30: European Organization for Research Treatment of Cancer Core Quality of Life Questionnaire. Item #30 “Global Quality of Life” is rated on an ordinal scale ranging from 1 (very poor) to 7 (excellent). The EORTC-scale categories 1, 2, and 7 are excluded due to very low numbers to fit in the statistical model.

**Table 4:** Difference between association values of self-reported and observed ADL abilities and EORTC QoL.

ADL-I (middle), *z*-score (*P* value)^∗^	AMPS motor (middle)	AMPS process (middle)
	0.316 (0.75)	0.286 (0.77)

ADL-I (high), *z*-score (*P* value)^∗^	AMPS motor (high)	AMPS process (high)
	0.585 (0.56)	0.518 (0.60)

ADL-I: ADL Interview; AMPS: Assessment of Motor and Process Skills. ^∗^The presented estimates are based on *z*-tests for differences between two odds ratios (OR) according to the ADL ability subgroups; middle and high and its impact on EORTC QoL, item #30 “Global Quality of Life” in the European Organization for Research Treatment of Cancer Core Quality of Life Questionnaire (EORTC-QLQ-C30). Level of significance: *P* < 0.05.

## Data Availability

The numerical data used to support the findings of this study have been deposited in the Department of Public Health, Research Unit for General Practice, The Research Initiative for Activity Studies and Occupational Therapy, University of Southern Denmark, Odense, Denmark. Due to ethical concerns, supporting data cannot be made openly available. Further information about the data and conditions for access are available upon request from the authors.

## References

[1] Townsend E. A., Polatajko H. J. (2007). *Enabling Occupation II: Advancing an Occupational Therapy Vision for Health, Well-Being, & Justice through Occupation*.

[2] Yerxa E. J. (1998). Health and the human spirit for occupation. *The American Journal of Occupational Therapy*.

[3] Kielhofner G., Kielhofner G. (2009). *Conceptual Foundations of Occupational Therapy Practice*.

[4] Yerxa E. J. (1990). An introduction to occupational science, a foundation for occupational therapy in the 21st century. *Occupational Therapy In Health Care*.

[5] Fisher A. G. (2009). *Occupational Therapy Intervention Process Model: A Model for Planning and Implementing Top-Down, Client-Centered and Occupation-Based Interventions*.

[6] Wæhrens E. E., Wæhrens E. E. (2015). ADL-begrebet. *Almidelig Daglig Levevis - ADL. (Activity of Daily Living – ADL)*.

[7] Armstrong D., Caldwell D. (2004). Origins of the concept of quality of life in health care: a rhetorical solution to a political problem. *Social Theory & Health*.

[8] Velikova G., Coens C., Efficace F. (2012). Health-Related Quality of Life in EORTC clinical trials — 30 years of progress from methodological developments to making a real impact on oncology practice. *European Journal of Cancer Supplements*.

[9] Pierce D. E., Pierce D. E. (2014). Relational research in occupational science. *Occupational Science for Occupational Therapy*.

[10] Hand C., Law M., McColl M. A. (2011). Occupational therapy interventions for chronic diseases: a scoping review. *The American Journal of Occupational Therapy*.

[11] Bourbeau J. (2009). Activities of life: the COPD patient. *COPD: Journal of Chronic Obstructive Pulmonary Disease*.

[12] Bendixen H. J., Ejlersen Wæhrens E., Wilcke J. T., Sørensen L. V. (2014). Self-reported quality of ADL task performance among patients with COPD exacerbations. *Scandinavian Journal of Occupational Therapy*.

[13] Mansson E., Lexell J. (2004). Performance of activities of daily living in multiple sclerosis. *Disability and Rehabilitation*.

[14] Moore A. R., Straube S., Paine J., Phillips C. J., Derry S., McQuay H. J. (2010). Fibromyalgia: moderate and substantial pain intensity reduction predicts improvement in other outcomes and substantial quality of life gain. *Pain*.

[15] Sundhedsstyrelsen (2012). *Forløbsprogram for rehabilitering og palliation i forbindelse med kræft: del af samlet forløbsprogram for kræft. (The Danish Health Autority: Guideline for rehabilitation and palliation for people with cancer: part of the overall guideline for cancer)*.

[16] Grønvold M. (2006). *Kræftpatientens verden: en undersøgelse af hvad danske kræftpatienter har brug for. (The Cancer Patients world: an ivestigation of what Danish cancer patients need)*.

[17] Johnsen A. T., Petersen M. A., Pedersen L., Groenvold M. (2009). Symptoms and problems in a nationally representative sample of advanced cancer patients. *Palliative Medicine*.

[18] Johnsen A. T., Petersen M. A., Pedersen L., Houmann L. J., Groenvold M. (2013). Do advanced cancer patients in Denmark receive the help they need? A nationally representative survey of the need related to 12 frequent symptoms/problems. *Psycho-Oncology*.

[19] Cheville A. L., Troxel A. B., Basford J. R., Kornblith A. B. (2008). Prevalence and treatment patterns of physical impairments in patients with metastatic breast cancer. *Journal of Clinical Oncology*.

[20] Rainbird K., Perkins J., Sanson-Fisher R., Rolfe I., Anseline P. (2009). The needs of patients with advanced, incurable cancer. *British Journal of Cancer*.

[21] Quach C., Sanoff H. K., Williams G. R., Lyons J. C., Reeve B. B. (2015). Impact of colorectal cancer diagnosis and treatment on health-related quality of life among older Americans: a population-based, case-control study. *Cancer*.

[22] Williams K., Jackson S. E., Beeken R. J., Steptoe A., Wardle J. (2016). The impact of a cancer diagnosis on health and well-being: a prospective, population-based study. *Psychooncology*.

[23] Elmqvist M. A., Jordhoy M. S., Bjordal K., Kaasa S., Jannert M. (2009). Health-related quality of life during the last three months of life in patients with advanced cancer. *Supportive Care in Cancer*.

[24] Solomon R., Kirwin P., Van Ness P. H., O'Leary J., Fried T. R. (2010). Trajectories of quality of life in older persons with advanced illness. *Journal of the American Geriatrics Society*.

[25] Yokoo M., Akechi T., Takayama T. (2014). Comprehensive assessment of cancer patients’ concerns and the association with quality of life. *Japanese Journal of Clinical Oncology*.

[26] Garrison C. M., Overcash J., McMillan S. C. (2011). Predictors of quality of life in elderly hospice patients with cancer. *Journal of Hospice & Palliative Nursing*.

[27] Nielsen K. T., Waehrens E. E. (2015). Occupational therapy evaluation: use of self-report and/or observation?. *Scandinavian Journal of Occupational Therapy*.

[28] Waehrens E. E., Bliddal H., Danneskiold-Samsoe B., Lund H., Fisher A. G. (2012). Differences between questionnaire- and interview-based measures of activities of daily living (ADL) ability and their association with observed ADL ability in women with rheumatoid arthritis, knee osteoarthritis, and fibromyalgia. *Scandinavian Journal of Rheumatology*.

[29] Wæhrens E. E., la Cour K., Brandt Å. (2014). Quality of ADL task performance based on self-report and observation in people living at home with cancer. *16th International Congress of the World Federation of Occupational Therapists*.

[30] Amris K., Wæhrens E. E., Stockmarr A., Bliddal H., Danneskiold-Samsøe B. (2014). Factors influencing observed and self-reported functional ability in women with chronic widespread pain: a cross-sectional study. *Journal of Rehabilitation Medicine*.

[31] Brandt A., Pilegaard M. S., Oestergaard L. G. (2016). Effectiveness of the “Cancer Home-Life Intervention” on everyday activities and quality of life in people with advanced cancer living at home: a randomised controlled trial and an economic evaluation. *BMC Palliative Care*.

[32] Brandt Å., la Cour K., Wæhrens E., Peoples H. (2016). Living at home with advanced cancer: what people do and how they manage their everyday activities. *The American Journal of Occupational Therapy*.

[33] Aaronson N. K., Ahmedzai S., Bergman B. (1993). The European Organization for Research and Treatment of Cancer QLQ-C30: a quality-of-life instrument for use in international clinical trials in oncology. *Journal of the National Cancer Institute*.

[34] Fayers P., Aaronson N. K., Bjordal K., Groenvold M., Curran D., Bottomley A. (2001). *EORTC QLQ-C30 Scoring Manual*.

[35] Groenvold M., Klee M. C., Sprangers M. A. G., Aaronson N. K. (1997). Validation of the EORTC QLQ-C30 quality of life questionnaire through combined qualitative and quantitative assessment of patient-observer agreement. *Journal of Clinical Epidemiology*.

[36] Smith A. B., Cocks K., Taylor M., Parry D. (2014). Most domains of the European Organisation for Research and Treatment of Cancer Quality of Life Questionnaire C30 are reliable. *Journal of Clinical Epidemiology*.

[37] Kaasa S., Bjordal K., Aaronson N. (1995). The EORTC core quality of life questionnaire (QLQ-C30): validity and reliability when analysed with patients treated with palliative radiotherapy. *European Journal of Cancer*.

[38] Coates A., Porzsolt F., Osoba D. (1997). Quality of life in oncology practice: prognostic value of EORTC QLQ-C30 scores in patients with advanced malignancy. *European Journal of Cancer*.

[39] Wæhrens E. E. (2010). *Measuring Quality of Occupational Performance Based on Self-Report and Observation: Development and Validation of Instruments to Evaluate ADL Task Performance*.

[40] Wæhrens E. E., Brandt Å., Peoples H., la Cour K. (2019). *Everyday Activities when Living Home with Advanced Cancer: A Cross-Sectional Study*.

[41] Fisher A. G., Jones K. B. (2012). *Assessment of Motor and Process Skills Volume I - Development, Standardization, and Administration Manual*.

[42] Fisher A. G., Jones K. B. (2014). *Assesment of Motor and Process Skills: User Manual, vol. 2*.

[43] AMPS (2010). *Computer-Scoring Software*.

[44] Gerber L. H., Hoffman K., Chaudhry U. (2006). Functional outcomes and life satisfaction in long-term survivors of pediatric sarcomas. *Archives of Physical Medicine and Rehabilitation*.

[45] Parks R., Rasch E. K., Mansky P. J., Oakley F. (2009). Differences in activities of daily living performance between long-term pediatric sarcoma survivors and a matched comparison group on standardized testing. *Pediatric Blood & Cancer*.

[46] World Medical Associations WMA declaration of Helsinki – Ethical principles for medical research involving human subjects. https://www.wma.net/policies-post/wma-declaration-of-helsinki-ethical-principles-for-medical-research-involving-human-subjects/.

[47] Stata (2015). *Statistics/Data Analysis*.

[48] Kirkwood B. R., Sterne J. A. C. (2003). *Essential Medical Statistics*.

[49] Rabe-Hesketh S., Everitt B. S. (2007). *A Handbook of Statistical Analyses Using Stata*.

[50] Merritt B. K. (2011). Validity of using the Assessment of Motor and Process Skills to determine the need for assistance. *The American Journal of Occupational Therapy*.

[51] Merritt B. K. (2010). Utilizing AMPS ability measures to predict level of community dependence. *Scandinavian Journal of Occupational Therapy*.

[52] Thome B., Hallberg I. R. (2004). Quality of life in older people with cancer – a gender perspective. *European Journal of Cancer Care*.

[53] Lundh Hagelin C., Seiger A., Furst C. J. (2006). Quality of life in terminal care—with special reference to age, gender and marital status. *Supportive Care in Cancer*.

[54] King M. T., Kenny P., Shiell A., Hall J., Boyages J. (2000). Quality of life three months and one year after first treatment for early stage breast cancer: influence of treatment and patient characteristics. *Quality of Life Research*.

[55] Ganz P. A., Jack Lee J., Siau J. (1991). Quality of life assessment. An independent prognostic variable for survival in lung cancer. *Cancer*.

[56] Fletcher R. H., Fletcher S. W., Fletcher G. S. (2014). *Clinical Epidemiology: The Essentials*.

[57] Burkhardt A., Ivy M., Kannenberg K. R., Low J. F., Marc-Aurele J., Youngstrom L. M. J. (2011). The role of occupational therapy in end-of-life care. *The American Journal of Occupational Therapy*.

[58] Crompton S. (2004). *Occupational Therapy Intervention in Cancer. Guidance for Professionals, Managers and Decision-Makers*.

[59] Cooper J. (2006). *Occupational Therapy in Oncology and Palliative Care*.

